# The Soluble Form of Toll-Like Receptor 2 Is Elevated in Serum of Multiple Sclerosis Patients: A Novel Potential Disease Biomarker

**DOI:** 10.3389/fimmu.2018.00457

**Published:** 2018-03-14

**Authors:** Md Jakir Hossain, Elena Morandi, Radu Tanasescu, Nanci Frakich, Marzia Caldano, David Onion, Tola A. Faraj, Clett Erridge, Bruno Gran

**Affiliations:** ^1^Division of Clinical Neuroscience, University of Nottingham School of Medicine, Queen’s Medical Centre, Nottingham, United Kingdom; ^2^Department of Neurology, Neurosurgery and Psychiatry, University of Medicine and Pharmacy Carol Davila, Colentina Hospital, Bucharest, Romania; ^3^Neurologia – Centro Riferimento Regionale Sclerosi Multipla (CReSM), Neuroscience Institute Cavalieri Ottolenghi (NICO), San Luigi University Hospital, Orbassano, Turin, Italy; ^4^School of Life Sciences, University of Nottingham Flow Cytometry Facility, University of Nottingham, Nottingham, United Kingdom; ^5^Department of Cardiovascular Sciences, University of Leicester, Clinical Sciences Wing, Glenfield General Hospital, Leicester, United Kingdom; ^6^Department of Biomedical and Forensic Sciences, Anglia Ruskin University, Cambridge, United Kingdom; ^7^Department of Neurology, Nottingham University Hospitals NHS Trust, Nottingham, United Kingdom

**Keywords:** toll-like receptor 2, soluble TLR2, multiple sclerosis, urinary tract infection, biomarker

## Abstract

Multiple sclerosis (MS) is an immune-mediated inflammatory demyelinating disease of the central nervous system. It was previously shown that toll-like receptor (TLR)-2 signaling plays a key role in the murine experimental autoimmune encephalomyelitis (EAE) model of MS, and that TLR2-stimulation of regulatory T cells (Tregs) promotes their conversion to T helper 17 (Th17) cells. Here, we sought potential sources of TLR2 stimulation and evidence of TLR2 activity in MS patient clinical samples. Soluble TLR2 (sTLR2) was found to be significantly elevated in sera of MS patients (*n* = 21), in both relapse and remission, compared to healthy controls (HC) (*n* = 24). This was not associated with the acute phase reaction (APR) as measured by serum C-reactive protein (CRP) level, which was similarly increased in MS patients compared to controls. An independent validation cohort from a different ethnic background showed a similar upward trend in mean sTLR2 values in relapsing-remitting MS (RRMS) patients, and significant differences in sTLR2 values between patients and HC were preserved when the data from the two cohorts were pooled together (*n* = 41 RRMS and 44 HC, *P* = 0.0006). TLR2-stimulants, measured using a human embryonic kidney (HEK)-293 cells transfectant reporter assay, were significantly higher in urine of MS patients than HC. A screen of several common urinary tract infections (UTI)-related organisms showed strong induction of TLR2-signaling in the same assay. Taken together, these results indicate that two different markers of TLR2-activity—urinary TLR2-stimulants and serum sTLR2 levels—are significantly elevated in MS patients compared to HC.

## Introduction

Multiple sclerosis (MS) is a chronic immune-mediated inflammatory disease of the central nervous system (CNS) in which autoreactive lymphocytes infiltrate the blood–brain barrier to target axonal myelin damage leading to neurological complications (1). MS can have diverse forms of clinical course; the most common form at presentation is the relapsing-remitting (RR) MS, manifesting as recurrent attacks (relapses) of neurological dysfunction followed by periods of remission. T cells are known to play a central role in the pathogenesis of both MS and its animal model experimental autoimmune encephalomyelitis (EAE) and, therefore, T cells remain the prime target of effective immunomodulatory and immunosuppressive treatments in MS (2, 3).

Toll-like receptors (TLRs) are a family of innate immune receptors that serve to trigger inflammatory signaling through their detection of exogenous pathogen-associated molecular patterns (PAMPs) and endogenous danger associated molecular patterns. TLRs are known to play critical roles in both MS and EAE by influencing the initiation of the disease, the triggering of relapses, and regulation of CNS damage (4). TLR2 is reported to be expressed on many different cell types of the innate and adaptive immune systems, and also in the CNS (5, 6). Due to its ability to form heterodimers with either TLR1 or TLR6, TLR2 can sense a broad spectrum of microbial as well as endogenous ligands indicating its importance both in infection and autoimmunity (7–9).

Although the etiology of MS is complex, both genetics and environmental factors are known to contribute to risk of disease (10). Infections are well recognized environmental triggers for MS susceptibility, pathogenesis, and exacerbation (11–13). For example, upper respiratory tract and urinary tract infections (UTI) are considered to be important triggers for the activation of inflammatory relapse in MS (14, 15). However, it remains unclear how bacterial infections may trigger exacerbations.

Previous results from our lab have shown that TLR2 stimulation of human regulatory T cells (Tregs), which have impaired suppressive function in MS patients (16, 17), leads to reduced Treg function and drives their differentiation toward an inflammatory Th17-like phenotype (18). Moreover, Tregs from RRMS patients were more susceptible to such TLR2-induced effects compared to healthy controls (HC) (19). Thus, we hypothesized that exposure to stimulants of TLR2-signaling *in vivo* could shift the Treg/Th17 balance toward a proinflammatory state in MS, promoting disease activity and progression.

One of the major negative regulators of TLR-signaling is the generation of extracellular soluble TLRs, which serve as decoy receptors to limit ligand-induced signaling (20). In the human immune system, the soluble TLR2 (sTLR2) is reported to suppress TLR2-mediated inflammation, in part by preventing its binding to the co-receptor CD14 (21–23). sTLR2 may be produced by protease cleavage, or by ectodomain shedding (24, 25), resulting in at least six distinct sTLR2 polypeptides, which have been identified in human breast milk, plasma, and monocyte culture supernatant (23). Very recent data show that the cysteine protease calpain, which is released by activated T cells, generates sTLR2 by cleavage of surface TLR2 in its transmembrane domain (26). sTLR2 can also be released from cells in response to TLR2-stimulation, suggesting that it could serve as a marker of recent TLR2-signaling, or other inflammatory activity (25).

In this study, we sought evidence of recent exposure to TLR2-stimulation in MS patient clinical samples. sTLR2 levels were found to be significantly higher in sera of MS patients compared to HC, and TLR2-stimulants were present at significantly higher levels in urine of MS patients compared to HC, consistent with robust TLR2-activation by a panel of common UTI-relevant organisms. These results offer the first evidence that MS patients may be at risk of increased exposure to TLR2-ligands compared to healthy subjects.

## Materials and Methods

### Ethics Statement

This study was carried out in accordance with the recommendations of Nottingham Research. Ethics Committee 2 with written informed consent from all subjects. All subjects gave written informed consent in accordance with the Declaration of Helsinki. The protocol was approved by the Nottingham Research Ethics Committee 2 (REC Reference 08/H0408/167).

### Study Participants

Multiple sclerosis patients attending outpatient clinics at Nottingham University Hospitals were recruited for this study. The study included 35 adult MS patients with clinically definite MS, according to the McDonald Criteria (27), aged 17–57 years (mean: 40.45 ± 10.6). 18 patients had not been treated with any disease-modifying treatments (DMTs), 6 were taking interferon-β, 4 were on Glatiramer acetate, 3 were on Fingolimod, 2 were receiving Dimethyl fumarate, and 2 were receiving corticosteroids. In addition, 25 HC aged 26–58 years (mean: 40.73 ± 10.2) were recruited. A relapse was defined as patient-reported symptoms or objectively observed signs typical of an acute inflammatory demyelinating event in the CNS, lasting at least 24 h (27). HC had no history of autoimmune disease or recent symptomatic infections. There was no significant age difference between MS patients and HC (*P* = 0.153). All MS patients and HC gave written informed consent prior to blood and urine sampling. The study was approved by the Nottingham Research Ethics Committee and by Nottingham University Hospitals National Health Service Trust Research and Innovation Services. Age and sex-matched samples for the validation cohort were received from University Hospital San Luigi Gonzaga, Orbassano, Turin, Italy which included 20 adult RRMS patients aged between 21 and 55 years (mean 33.35 ± 10.74) and 20 HC aged between 22 and 57 years (mean 33.6 ± 10.97). All the samples in the validation cohort were treatment naïve and there was no age difference between RRMS and HC (*P* = 0.942). Both patients and controls were Caucasians, mainly from the Piedmont region. Demographic data for the two cohorts are presented in Table 1.

**Table 1:** Baseline demographics and clinical data of patients and HC.

	Discovery cohort		Validation cohort	
	MS (*n* = 35)	HC (*n* = 25)	MS (*n* = 20)	HC (*n* = 20)
Age (mean ± SD)	40.45 ± 10.6	40.73 ± 10.2	33.35 ± 10.74	33.6 ± 10.97
Age range	17–57	26–58	21–55	22–57
*P* value	0.153 (ns)		0.942 (ns)	
Male	11	11	5	5
Female	24	14	15	15
MS course	RRMS (*n* = 35)	–	RRMS (*n* = 20)	–
Disease duration in year from CIS (mean ± SEM)	8.79 ± 1.3	–	4.18 ± 1.85	–
Disease duration in year from CD-MS (mean ± SEM)	5.99 ± 0.99	–	0.36 ± 0.16	–
EDSS (mean ± SEM)	N/A	–	0.97 ± 0.21	–

*ns, not significant; RRMS, relapsing-remitting MS, CIS, clinically isolated syndrome; CD-MS, clinically definite MS; EDSS; expanded disability status score; N/A, not available*.

*Disease duration was calculated from the CIS or CDMS date to the sampling date. EDSS in the discovery cohort were only available for 4 patients. EDSS in the validation cohort were available for 19 out of 20 patients*.

### Enzyme-Linked Immunosorbent Assay (ELISA)

Soluble TLR2 and sTLR4 were measured in serum and urine samples by ELISA (DTLR20, R&D Systems, UK, and SEA753Hu; Cloud-Clone Corp., USA, respectively). For the validation cohort sTLR2 was measured using a different ELISA kit (SEA753Hu; Cloud-Clone Corp., USA). CRP was measured in serum samples by high-sensitivity ELISA (DY1707, R&D Systems).

### Determination of TLR2 Stimulants

TLR2 stimulants in urine and serum samples from MS patients and HC were measured using a human embryonic kidney (HEK)-293 cell TLR-transfection assay, as described previously (28). Briefly, cells were transiently transfected with TLR2, CD14, nuclear factor kappa-light-chain-enhancer of activated B cells (NF-κB)-reporter, and thymidine-kinase promoter-driven reporter constructs, then challenged 3 days later with indicated concentrations of defined TLR-ligands, volunteer serums, heat-killed urine samples, or heat-killed bacteria. 18 h later, NF-κB-dependent reporter expression was measured by luminometry and normalized to co-transfected renilla reporter. A standard curve was prepared on each plate by plotting fold NF-κB induction vs concentration using the defined TLR2-ligand Pam_3_CSK_4_. Because it has been proposed that diverse ligands beyond lipopeptides may stimulate TLR2, the TLR2-stimulating capacity of the samples are presented as biological activities relative to this standard curve and are expressed as ng/ml Pam_3_CSK_4_-equivalents or bacterial lipopeptide equivalent (BLP) equivalents.

### TLR2 Stimulating Activity in Heat-Killed Bacteria and *Helicobacter pylori* Lysate

A selection of eight representative UTI-related Gram-positive and Gram-negative bacteria was tested using the same HEK-293-TLR2 transfection system to determine their capacity to stimulate TLR2. All bacteria examined were of hazard group 2 or lower and were studied in a class 2 containment facility. A whole bacterial cell lysate was also prepared from *H. pylori* (SS1 strain) for assay in the same system. To prepare the lysate, *H. pylori* cultures from 10 blood agar base 2 plates (Oxoid) cultured for 24 h under microaerobic conditions at 37°C were harvested into ice-cold sterile phosphate-buffered saline. This suspension was then disrupted using a Soniprep 150 sonicator (SANYO, Watford, UK), with 6 × 10s bursts at amplitude of 10 µm, then aliquoted and stored at −20°C. There were approximately 6 × 10^9^ bacteria per ml before sonication, corresponding to 1 × 10^7^ bacteria per ml when diluted, and the protein content of the lysate was 13.8 mg/ml as determined using the BCA protein assay kit (Thermo). The TLR2-stimulating capacities of bacteria and lysates in these experiments are presented as NF-κB fold induction vs cells cultured in medium alone.

### Dipstick Test

Urine samples were tested using Combur-Test Strip dipsticks (Cobas, Roche, Switzerland). Positive results for leukocytes, nitrite, protein, and erythrocytes were considered potential signs of infection.

### Flow Cytometry

Flow cytometric analyses were carried out using frozen peripheral blood mononuclear cells (PBMC) from RRMS patients collected during relapse and remission. After thawing, the cells were cultured for 5 h (short-term stimulation) with phorbol 12-myristate 13-acetate (0.1 µg/ml), ionomycin (1 µg/ml), and Brefeldin-A (10 µg/ml, added only in the last hour). After stimulation and washing, cells were stained with Live/Dead^®^ Fixable Blue Dead Cell Stain Kit (ThermoFisher Scientific) for 30 min at room temperature. Cells were then stained extracellularly with anti-CD4 (BD Pharmingen) and anti-TLR2 (eBioscience, Clone TL2.1). Cells were acquired using an LSR II flow cytometer (BD Biosciences), collecting a minimum of 50,000 events in each sample, and analyzed using Kaluza software (Beckman Coulter, version 1.5). Gating strategy ungated PBMCs were plotted on SSC/FSC plot and then lymphocytes were gated by excluding dead cells/debris. Then, from the lymphocytes, live cells were gated. Then, the percentage of live lymphocytes that were CD4+/TLR2+ were selected.

### Calpain Treatment

Peripheral blood mononuclear cells from healthy volunteers were treated with 4 µg/ml of Calpain (Natural human Calpain 1 protein, AbCam, Product no. ab91019) for 1 h at 37°C and then stained with anti-CD4 and anti-TLR2 antibodies and analyzed by flow cytometry as above. Gating strategy ungated PBMCs were plotted on SSC/FSC plot and then lymphocytes were gated by excluding dead cells/debris. Then, from the lymphocytes, CD4+ cells were gated and then percentage of TLR2+ cells from CD4+ population were identified.

### Statistical Analyses

The data are presented as mean (±SD or SEM). All statistical analyses were performed using Prism 7.01 (GraphPad Software, San Diego, CA, USA). Data which were not normally distributed were log transformed before comparison of groups using the Mann–Whitney *U* test and Wilcoxon matched-pairs signed rank test (for paired samples). *p* Values <0.05 were considered significant.

## Results

### Levels of sTLR2 and C-Reactive Protein (CRP) Are Significantly Elevated in Sera of RRMS Patients

**Figure 1:**
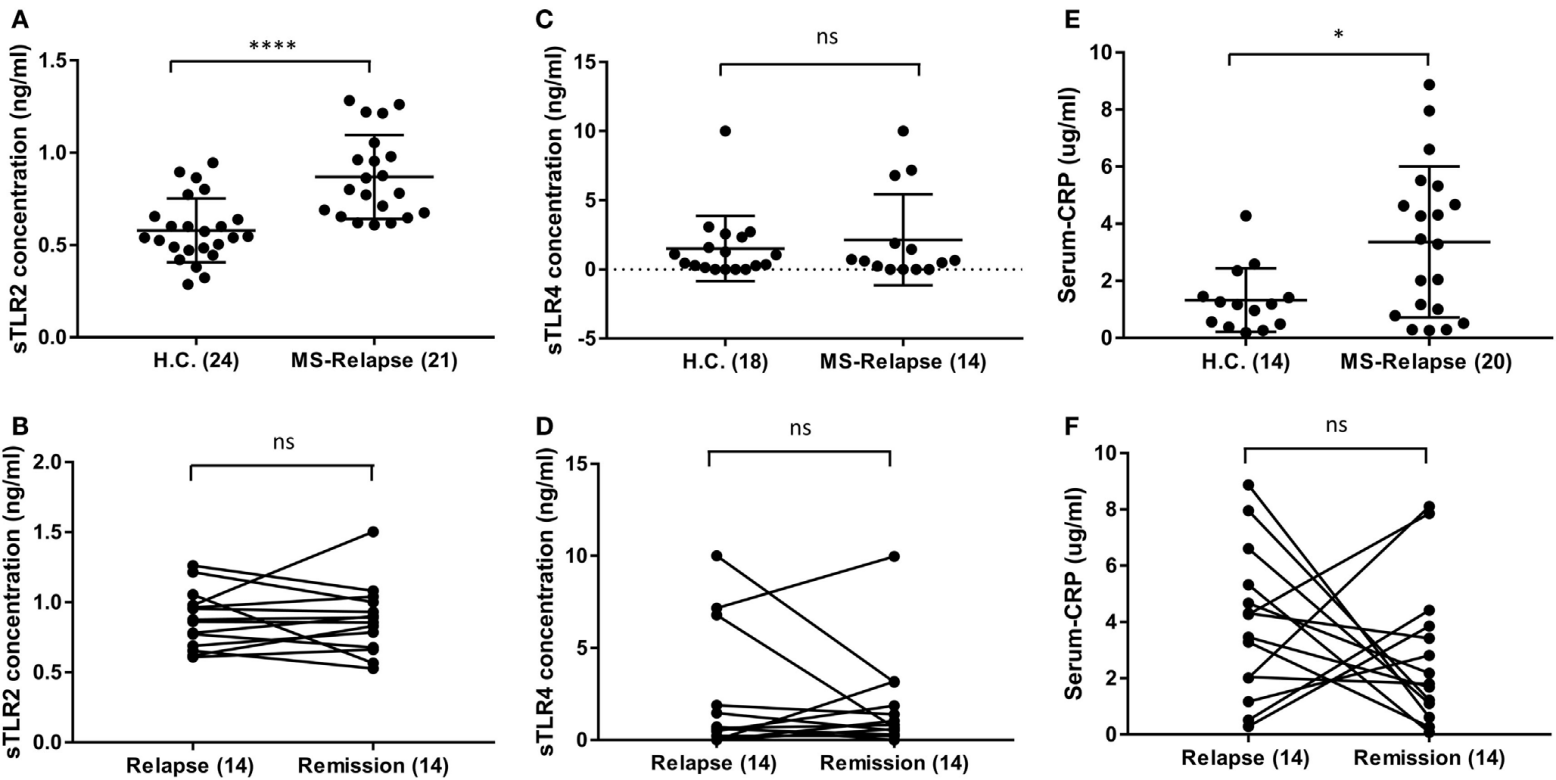
Level of soluble TLR2 (sTLR2), sTLR4, and hsC-reactive protein (CRP) measured by enzyme-linked immunosorbent assay in the sera of relapsing-remitting (RR) MS (RRMS) patients and compared with HC. The data are presented as mean ± SD. **(A)** sTLR2 measured in the serum samples from RRMS patients during relapse (*n* = 21) and compared with HC (*n* = 24). Mann–Whitney test showed significantly higher sTLR2 values during relapse (*P* < 0.0001). Significantly higher sTLR2 values were also observed during remission (*n* = 21) compared to HC (*P* = 0.0002) (not shown). **(B)** Wilcoxon matched-pairs signed rank test between paired relapse and remission samples showed no significant difference (*n* = 14, *P* = 0.9032). **(C)** sTLR4 measured in the serum samples from RRMS patients during relapse (*n* = 14) and compared with HC (*n* = 18). Mann–Whitney test showed no significant differences (*P* = 0.9926). Comparison between remission (14) and HC also showed no significant difference (*P* = 0.0860) (not shown). **(D)** Wilcoxon matched-pairs signed rank test between paired relapse and remission samples showed no significant difference (*n* = 14, *P* > 0.9999). **(E)** Serum CRP measured in the samples from RRMS patients during relapse (*n* = 20) and HC (*n* = 14). Mann–Whitney test showed significantly higher CRP during relapse (*P* = 0.0352) compared to HC. No significant difference observed between HC vs remission samples (not shown). **(F)** Wilcoxon matched-pairs signed rank test between paired relapse and remission samples showed no significant difference (*n* = 14, *P* = 0.4263). * *P* < 0.05, **** *P* < 0.0001, ns, not significant.

As it has been reported that serum sTLR2 is a biomarker of recent infection, and of disease activity in other conditions, we measured sTLR2 levels in serum samples from MS patients in relapse and remission and compared them with those from HC (29, 30). We observed significantly higher levels of sTLR2 in the serum samples of MS patients both during relapse (0.87 ng/ml) and remission (0.85 ng/ml) when compared with HC (0.58 ng/ml) (Figure 1A). However, there was no significant difference in the sTLR2 values between paired MS relapse and remission samples (*n* = 14, Figure 1B). Since, both TLR2 and TLR4 expression has been reported to be elevated in the PBMCs from MS patients and soluble forms for both of these receptors (sTLR2 and sTLR4) were detected in various infectious and non-infectious inflammatory conditions, we also measured sTLR4 levels in the serum samples (31, 32). We found that sTLR4 levels were not significantly different between groups (1.52, 2.14, and 1.69 ng/ml) for HC, MS relapse, and MS remission, respectively (Figures 1C,D). Serum samples were also tested for the presence of TLR2 stimulants, but these were below the limit of detection by the HEK293-TLR2 transfection assay (data not shown).

Similar to sTLR2, we found significantly higher levels of serum CRP during MS relapse compared to HC, although, there was no significant difference between paired relapse and remission samples (Figures 1E,F). This raised the possibility that sTLR2 may be increased in relation to the acute phase reaction (APR). However, there was no significant correlation between serum levels of CRP and sTLR2 or sTLR4, and an *in vitro* model of the APR (interleukin-1β treated hepatocarcinoma cell line cells), revealed that sTLR2 is not released by stimulated hepatocytes (data not shown). Together, these findings suggest that the observed increase in serum sTLR2 in MS patients is likely not directly caused by the APR.

### Serum sTLR2 Is Not Significantly Elevated in Validation Cohort but Significant in the Pooled Data

**Figure 2:**
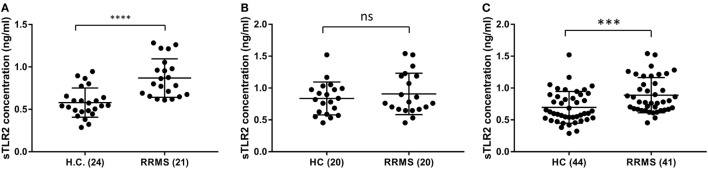
Comparison of serum soluble TLR2 (sTLR2) levels in the discovery cohort, validation cohort, and pooled data measured by enzyme-linked immunosorbent assay and analysis of pooled data. The data are presented as mean ± SD. **(A)** sTLR2 measured in the serum samples from RRMS patients and HC in the discovery cohort (*n* = 21 RRMS, 24 HC). Mann–Whitney test showed significantly higher sTLR2 values in the RRMS patients (*P* < 0.0001). **(B)** sTLR2 levels measured in the serum samples from RRMS patients and HC in the validation cohort (*n* = 20 RRMS, 20 HC). Mann–Whitney test showed no significant difference between RRMS patients and HC (*P* = 0.6061). **(C)** sTLR2 levels in the serum samples were pooled together from two cohorts and compared between patients and HC (*n* = 41 RRMS, 44 HC). Mann–Whitney test showed highly significant difference between RRMS patients and HC (*P* = 0.0006).

To validate the sTLR2 elevation in the serum of RRMS patients, we performed ELISA tests in the sera of an independent cohort of patient and HC from a different population (Piedmont, North-Western Italy). There was a similar upward trend of mean sTLR2 levels in the serum samples of RRMS patients (0.907 ng/ml) compared to HC (0.836 ng/ml) and the values were almost in the same concentration range as in the discovery data set although it did not reach statistical significance (*P* = 0.6061) (Figures 2A,B). We, therefore, pooled the data from our original discovery cohort and the validation cohort to see the overall trend. In the pooled data, sTLR2 levels were 0.888 and 0.696 ng/ml in the RRMS (*n* = 41) and HC (*n* = 44), respectively, and the analysis retained the original significance (*P* = 0.0006, Mann–Whitney test) (Figure 2C). We also measured the levels of sTLR4 in the serum samples from the validation cohort and the trend was the same as observed in our discovery cohort, with values that were very low or below the detection limit (data not shown).

### TLR2 Surface Expression on CD4+ T Cells Does Not Differ between MS Relapse and Remission, but Is Reduced by Calpain Treatment

**Figure 3:**
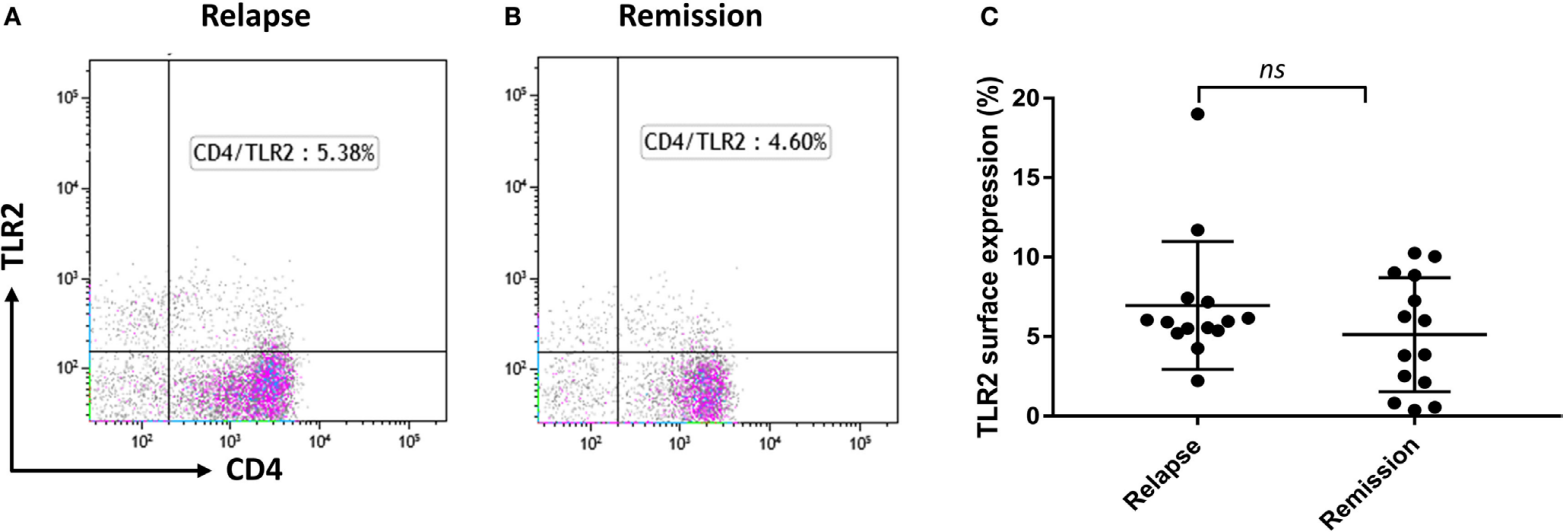
TLR2 surface expression in relapsing-remitting MS patients and effect of Calpain on TLR2 expression. **(A,B)** Representative flow cytometry dot plots showing percentage of CD4+ TLR2+ cells for the same patient during relapse and remission. Cells were gated on lymphocytes then live cells and then double gated for CD4 and TLR2. **(C)** Graphs showing the percentage of CD4+ TLR2+ lymphocytes comparing between relapse and remission in paired samples (*n* = 14). Data are presented as mean ± SD. Wilcoxon matched-pairs signed rank test gave the *P* value of 0.1937 showing no significant difference in TLR2 surface expression between relapse and remission.

As we reported recently that Tregs from RRMS patients are more susceptible to TLR2-stimulation than those of HC (19), we explored whether CD4+ T cells may have potential to contribute to the circulating pool of sTLR2. The percentage of CD4+ T cells surface expression of TLR2 was not significantly different between relapse and remission (Figures 3A–C). However, previous studies have shown that extracellular calpain, a cysteine protease released by T lymphocytes, has the ability to cleave TLR2, leading to increased levels of sTLR2 in the extracellular environment both in mice and humans (26). Treatment with exogenous calpain led to a non-significant reduction in TLR2 surface expression on CD4+ T cells (data not shown). However, we were unable to confirm this trend by ELISA, as sTLR2 levels were below the limit of detection in the supernatants of T cells cultured in the presence of calpain (data not shown).

### TLR2 Stimulant Activity but Not sTLR2 or sTLR4 Are Significantly Raised in the Urine of MS Patients Compared to Controls

**Figure 4:**
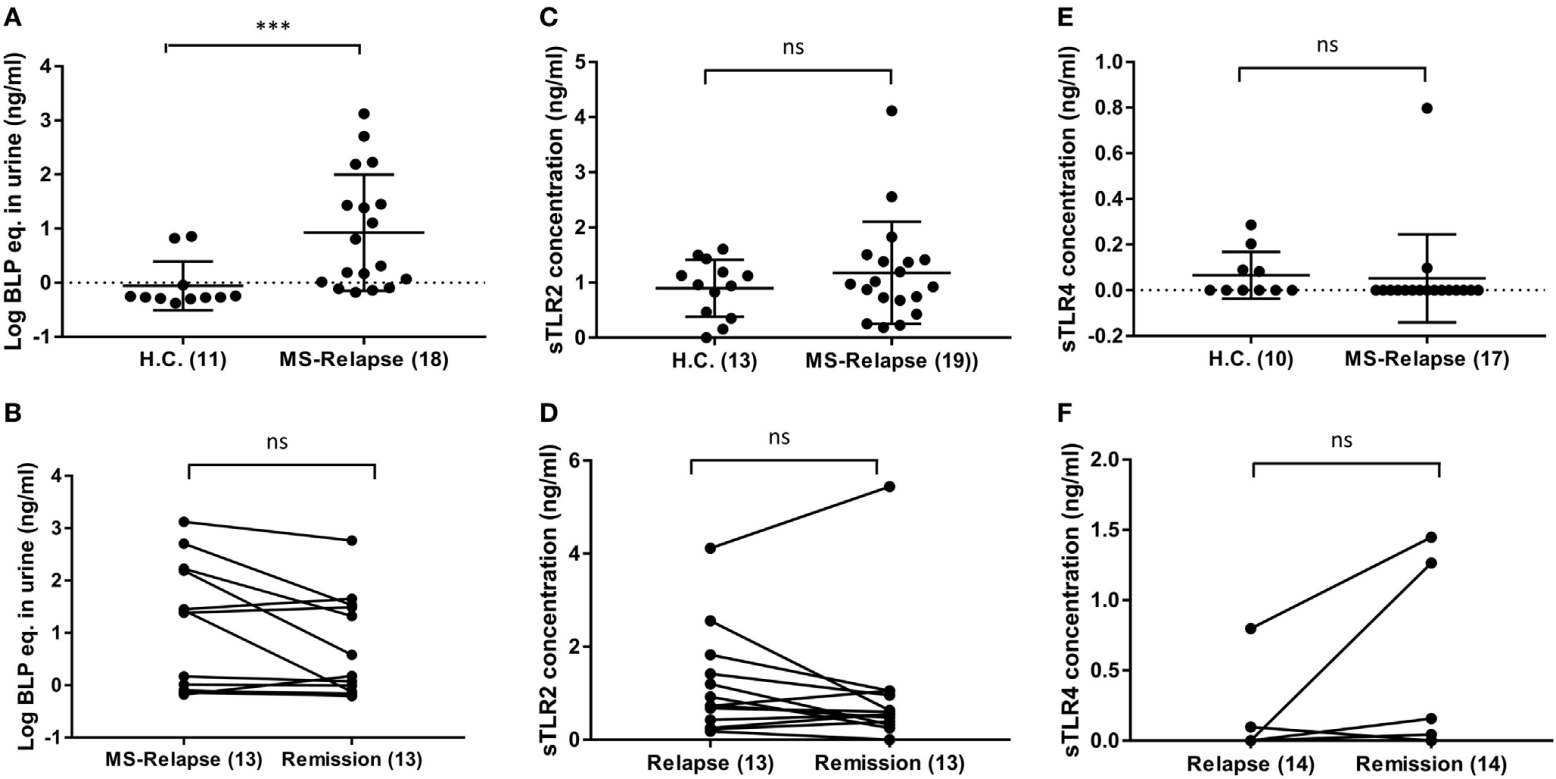
Level of soluble TLR2 (sTLR2), sTLR4, and TLR2 stimulants in the urine of relapsing-remitting (RR) MS (RRMS) patients compared with HC. sTLR2 and sTLR4 were measured by enzyme-linked immunosorbent assay (ELISA) and urinary TLR2 stimulants were measured in human embryonic kidney (HEK)-293-TLR2 transfection system. **(A)** TLR2 stimulants in the urine of RRMS patients were measured in HEK-293-TLR2 transfectant system during relapse (*n* = 18) and compared with HC (*n* = 11). Mann–Whitney test showed a significantly higher TLR2 stimulants in the urine of MS patients during relapse (*P* = 0.0004) compared with HC. Significantly higher urinary TLR2 stimulants were also observed during remission (*n* = 13) compared to HC (*P* = 0.0020) (not shown). **(B)** Wilcoxon matched-pairs signed rank test between relapse and remission also showed no significant difference (*n* = 13, *P* = 0.0803). The values are expressed as ng/ml of the bacterial lipopeptide equivalent and the data are presented as mean ± SD. The data in Figure 3 **(A,B)** are log transformed from original values. **(C)** sTLR2 measured in the urine samples from RRMS patients during relapse (*n* = 19) and HC (*n* = 13). The data are presented as mean ± SD. Mann–Whitney test showed no significant differences between HC vs relapse (*P* = 0.6498). HC vs remission (*n* = 20) also showed no significant difference (*P* = 0.2383) (not shown). **(D)** Wilcoxon matched-pairs signed rank test showed no significance between relapse and remission (*P* = 0.3054). **(E)** sTLR4 measured in the urine samples from RRMS patients during relapse (*n* = 17) and HC (*n* = 10). The data are presented as mean ± SD. Mann–Whitney test showed no significant differences between HC vs relapse (*P* = 0.1406). HC vs remission (*n* = 14) also showed no significant difference (*P* = 0.9290) (not shown). **(F)** Wilcoxon matched-pairs signed rank test showed no significance between relapse and remission (*n* = 14, *P* = 0.1875). *** *P* < 0.001. ns, not significant.

Soluble TLR2 may be released in response to excessive TLR2-stimulation (25) and so we sought evidence of potential sources of TLR2-stimulants that may be of relevance to MS. As UTI is a common complication of MS (33), we used urine dipstick tests to seek evidence of UTI in MS patients. Although levels of protein and erythrocytes were significantly increased in urine of MS patients compared to HC (Table 2), there were no significant differences in overall positivity for markers of infection between the groups, using this method. To gain further insight into whether MS patients may be exposed to increased TLR2-stimulants as a result of subclinical UTI not detectable by dipstick, we used a HEK293-TLR2 transfection assay calibrated with the synthetic ligand Pam_3_CSK_4_ to quantify the biological activities of TLR2-stimulants present in urine of HC and MS patients, as described previously (28). These measurements revealed that TLR2-stimulant activities were far greater in urine samples of MS patients compared to HC (1.72, 126.2, and 55.5 ng/ml), relative to Pam_3_CSK_4_-equivalent activity, for HC, MS relapse, and MS remission, respectively [*P* = 0.0004, *P* = 0.0020 (Figures 4A,B)].

**Table 2:** Results from urine dipstick tests.

Group	Leu	Nit	Prot	Eryt	Any (+ve)
Relapse (*n* = 19)	26%	21%	26%	26%	53%
Remission (*n* = 22)	14%	9%	23%	32%	41%
HC (*n* = 13)	8%	15%	0%	0%	23%
*P* value (Chi^2^ test HC vs all MS samples)	0.319	0.947	0.049	0.027	0.137

*Leu, leukocytes; Nit, nitrite; Prot, protein; Eryt, erythrocytes*.

Soluble TLR2 and sTLR4 levels were also measured in urine samples of HC and MS patients. Urinary sTLR2 levels were higher than measured in serum, but unlike serum sTLR2 levels, there were no statistically significant differences between HC and MS groups (Figures 4C,D). sTLR4 levels were below the limit of detection in most urine samples and the mean values were also not significantly different between the groups (Figures 4E,F).

### Diverse UTI-Related Bacteria Stimulate TLR2-signaling

**Figure 5:**
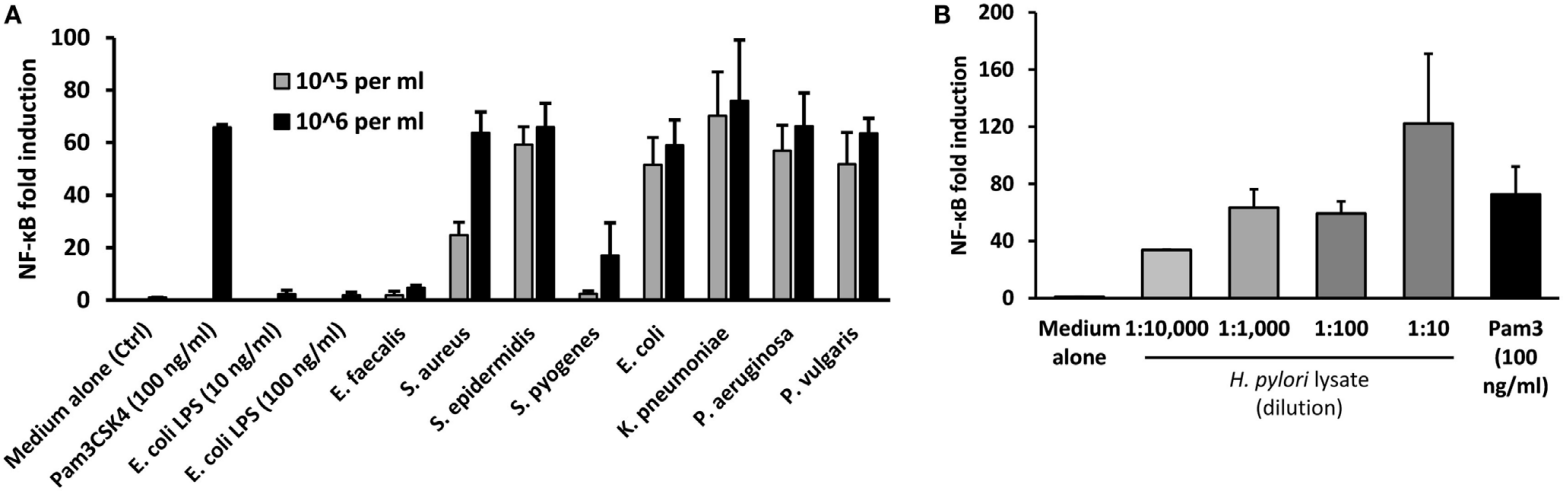
Stimulation of TLR2 signaling by urinary tract infections relevant heat-killed bacteria and *H. pylori* lysate. Pam3Cys (100 ng/ml) was used as the standard agonist for TLR2 and DMEM medium with 1% serum (D1) was used as control. *H. pylori* lysate also triggered robust TLR2-signaling in all four dilutions used, but one-way ANOVA did not reveal any dose effect (*P* = 0.0930). The results are expressed as NF-kB fold induction. **(A)** Effects of selected heat-killed bacteria on TLR2-signaling in human embryonic kidney (HEK)-293-TLR2 transfectants. The bacteria tested includes four Gram-positive (*E. faecalis, S. aureus, S. epidermidis, and S. pyogenes*) and four Gram-negative (*E. coli, K. pneumoniae, P. aeruginosa, and P. vulgaris*) species. Two different dilutions (10^6^ bacteria/ml and 10^5^ bacteria/ml) prepared in D1 was tested. The bars represent the results of three independent experiments expressed as mean ± SD. **(B)** Effect of *H. pylori* lysate on TLR2 signaling in HEK-293-TLR2 transfectants. Four different dilutions of the samples (1:10, 1:100, 1:1 K, 1:10 K) were tested. The bars represent the results of three independent experiments expressed as mean ± SEM. One-way ANOVA did not reveal any dose effect (*P* = 0.0930).

These findings suggested potential involvement of UTI-related bacteria in the stimulation of TLR2 in MS patients. To explore which types of bacteria may contribute to such activity, a panel of representative UTI-relevant bacteria was tested for their ability to trigger TLR2-signaling using the HEK293-TLR2 transfection system. All the Gram-negative organisms tested (*Escherichia coli, Klebsiella pneumoniae, Pseudomonas aeruginosa*, and *Proteus vulgaris*) were potent stimulators of TLR2-signaling (Figure 5A). However, while two of the Gram-positive organisms stimulated strong TLR2-signaling (*Staphylococcus aureus* and *Staphylococcus epidermidis*), two induced little or no TLR2-signaling (*Enterococcus faecalis* and *Streptococcus pyogenes*). As infection with the gastric pathogen *H. pylori* has also been associated with MS (34), the TLR2-stimulating capacity of *H. pylori* lysate was assessed, confirming that this organism also contains abundant TLR2-stimulants (Figure 5B). Thus, diverse bacteria responsible for infections which may be relevant to MS, of both Gram-positive and Gram-negative origin, are potent triggers of TLR2 signaling.

## Discussion

Increasing evidence supports a potential involvement of TLR2 in the development of MS. TLR2 is upregulated on PBMCs, Tregs, cerebrospinal fluid mononuclear cells, and in demyelinating lesions of MS patients [reviewed in Ref. (4)]. Experimental administration of defined bacterial ligands of TLR2, or the Gram-positive bacterium *Streptococcus pneumoniae*, increases clinical score in the murine EAE model of MS in a manner dependent on TLR2 (35, 36). Although experiments using mice globally deficient in TLR2 have yielded mixed results (4), adoptive transfer of TLR2-deficient T cells was shown to result in significantly reduced pathology in the passive EAE model, suggesting a key involvement of TLR2-signaling in T cells (37). Accordingly, we have shown that TLR2 stimulation of human Tregs impairs their suppressive function, and drives their differentiation toward an inflammatory Th17-like phenotype, particularly in cells from RRMS patients (18).

In this study, we sought evidence of TLR2 activity in MS clinical samples. We report, for the first time, that serum sTLR2 levels are significantly elevated in MS patients compared to match HC (Figure 1A). This is consistent with observations of increased serum sTLR2 in a number of other inflammatory diseases and disease models, including severe bacterial infection (38), experimental human endotoxemia (32), and the autoimmune disease systemic lupus erythematosus, where it serves as a biomarker of disease activity (29). Notably, sTLR2 can act as a decoy microbial receptor since it retains the capacity to bind with the PAMPs as well as TLR2 coreceptor CD14, thereby preventing surface TLR2 to bind with CD14 and PAMPs. The net effect is to reduce TLR2 activation (22). This has relevance for MS relapses, during which reduced TLR2 signaling might potentially attenuate inflammatory activity and clinical severity. When repeating sTLR measurement in an independent validation cohort, we found a trend toward higher sTLR2 levels in the RRMS patients compared to HC, with values in a similar concentration range as observed in our discovery dataset (Figures 2A,B). When we pooled the data from two cohorts, however, statistical differences between the RRMS (*n* = 41) and HC (*n* = 44) groups were highly significant (Figure 2C). The larger number of subjects studied in the pooled dataset gives us more assurance on the validity of the observation. To our knowledge, no previous study has reported sTLR2 levels in MS patients. We further analyzed the results to see whether treatments with DMTs had any influence on the serum sTLR2 levels and none was found either in the discovery cohort (*P* = 0.5201) or in the pooled data (*P* = 0.7587; all patients in the validation data were untreated).

The dominant cell-type responsible for increased sTLR2 release in MS remains to be clearly established. There was significant elevation in the levels of CRP between relapsing MS patients and HC indicative of chronic low-grade inflammation (Figure 1E) although no correlation between sTLR2 and hsCRP were observed which is consistent with a recent finding in diabetes (39). The lack of correlation with CRP, and its absence from stimulated hepatocyte cultures, suggests that it is likely not released by hepatocytes as part of the APR. We suspected that it may be released from CD4+ T cells, which express higher TLR2 in MS patients (30). However, sTLR2 released from CD4+ T cells treated with calpain (25) was not detectable, perhaps due calpain cleavage of epitopes required for binding by the antibodies used in the ELISA assay.

Although sTLR2 was readily detected in urine, urinary epithelial cells are also not likely the dominant source of serum sTLR2 in MS, since we saw no significant correlation between urinary and serum sTLR2 levels. There is recent evidence that nanovesicular exosomes derived from TLR-stimulated cells can communicate to other cells, reproduce a TLR-mediated response to infection, and modulate inflammation by preventing further binding of the TLR ligands (40). Further studies should investigate the content of exosomes derived from TLR2-stimulated cells as a potential source of sTLR2 and its possible effect on TLR2 signaling. More work will be required to establish which cell type is the dominant source of elevated sTLR2 in MS.

As sTLR2 is released in response to TLR2 overstimulation to downregulate inflammation (25, 32), we sought potential sources of TLR2-stimulants in MS clinical samples. UTIs are frequent in MS (33, 41), and there is evidence that disease exacerbations may be linked to recent UTI, particularly by Gram-negative bacteria (42, 43). Dipstick tests revealed no significant differences in frequency of indicators of infection between relapse and remission urine samples of our cohort. However, since dipstick tests have low sensitivity and lack reliability in diagnosing UTI (44, 45), we sought evidence of subclinical UTI using the more sensitive HEK-293-TLR2 transfection system, which can detect as few as 10^4^ bacterial cells/ml independently of bacterial viability (28).

This assay revealed that urine samples from MS patients in both relapse and remission contained a significantly greater content (~32–72 fold) of TLR2 stimulants than urine samples from HC (Figure 4A). Thus, subclinical UTI, at levels undetectable by dipstick test, may be more common in MS patients than previously thought (15, 44). Soluble TLRs, including sTLR2, are negative regulators of TLR signaling and function as a feedback mechanism to inhibit excessive TLR activation (20). In the context of this study, we hypothesized that overactivation of TLR2 by UTI-related organisms could induce intrinsic cellular processes mediated by proteolytic enzymes to cleave surface TLR2, release sTLR2, and downregulate TLR2 signaling. Although Gram-negative bacteria are commonly thought to be sensed principally *via* TLR4, we found that common UTI-relevant Gram-negative bacteria are also potent stimulators of TLR2 (Figure 5A). *H. pylori* lysate also triggered robust TLR2-signaling at all four dilutions tested (Figure 5B).

As infection with this organism is less commonly observed in MS patients, we and others have suggested that it may have protective properties against MS (34, 46). If this is the case, it is possible that the organism’s capacity to trigger TLR2-signaling is countered by the numerous modifiers of innate immune and T-lymphocyte function, particularly those resulting in increased Treg and reduced Th17 function, which *H. pylori* is established to secrete (47).

In conclusion, we report that two different markers of TLR2-activity—urinary TLR2-stimulants, and serum sTLR2 levels—are significantly elevated in MS patients compared to HC regardless of the treatment status. Although we saw no significant differences in these markers between relapse and remission samples in this study, this could be due to limited study power. Therefore, larger studies will be required to determine whether subclinical UTI is related to relapse in MS, and whether increased serum sTLR2 level could be used as a diagnostic marker for inflammation or disease exacerbation in MS.

## Ethics Statement

This study was carried out in accordance with the recommendations of Nottingham Research Ethics Committee 2 with written informed consent from all subjects. All subjects gave written informed consent in accordance with the Declaration of Helsinki. The protocol was approved by the Nottingham Research Ethics Committee 2 (REC Reference 08/H0408/167).

## Author Contributions

MH conducted the experiments, analyzed the data, and wrote the first draft of the paper. MH, EM, RT, CE, and BG all took part to conceive the study. DO helped in the flow cytometry. CE and TAF helped in the HEK-293 transfection experiment. CE performed the CRP assay and advised on the layout of the first draft. MC provided a well-characterized validation cohort of serum samples from people with RRMS and age- and gender-matched HC. NF performed ELISA experiments in the validation cohort. BG, RT, and CE critically edited the manuscript and BG supervised the study.

## Conflict of Interest Statement

The authors declare that the research was conducted in the absence of any commercial or financial relationships that could be construed as a potential conflict of interest. The reviewer MN and handling Editor declared their shared affiliation.

## Abbreviations

MS, multiple sclerosis; TLR, toll-like receptor; EAE, experimental autoimmune encephalomyelitis; Tregs, regulatory T cells; Th17, T helper 17 cells; sTLR2, soluble TLR2; APR, acute phase reaction; CRP, C-reactive protein; HEK, human embryonic kidney; UTI, urinary tract infections; CNS, central nervous system; RRMS, relapsing-remitting MS; PAMPs, pathogen-associated molecular patterns; DAMPs, danger associated molecular patterns; HC, healthy controls; DMTs, disease-modifying treatments; ELISA, enzyme-linked immunosorbent assay; CD, cluster of differentiation; NF-κB, nuclear factor kappa-light-chain-enhancer of activated B cells; BLP, bacterial lipopeptide equivalent equivalent; HP, *Helicobacter pylori*; PBS, phosphate-buffered saline; PBMC, peripheral blood mononuclear cells; PMA, phorbol 12-myristate 13-acetate; IL-1β, interleukin-1β; HepG2, hepatocarcinoma cell line.

## References

[1] Compston A Coles A. Multiple sclerosis. Lancet (2008) 372(9648):1502–17. 10.1016/S0140-6736(08)61620-7 18970977

[2] Constantinescu CS Gran B. The essential role of T cells in multiple sclerosis: a reappraisal. Biomed J (2014) 37(2):34–40. 10.4103/2319-4170.128746 24732657

[3] Fletcher J Lalor S Sweeney C Tubridy N Mills K T cells in multiple sclerosis and experimental autoimmune encephalomyelitis. Clin Exp Immunol (2010) 162(1):1–11. 10.1111/j.1365-2249.2010.04143.x 20682002 PMC2990924

[4] Miranda-Hernandez S Baxter AG. Role of toll-like receptors in multiple sclerosis. Am J Clin Exp Immunol (2013) 2(1):75–93. 23885326 PMC3714200

[5] Bsibsi M Ravid R Gveric D van Noort JM Broad expression of toll-like receptors in the human central nervous system. J Neuropathol Exp Neurol (2002) 61(11):1013–21. 10.1093/jnen/61.11.1013 12430718

[6] Flo TH Halaas Ø Torp S Ryan L Lien E Dybdahl B Differential expression of toll-like receptor 2 in human cells. J Leukoc Biol (2001) 69(3):474–81. 10.1189/jlb.69.3.474 11261796

[7] Borrello S Nicolo C Delogu G Pandolfi F Ria F. TLR2: a crossroads between infections and autoimmunity? Int J Immunopathol Pharmacol (2011) 24(3):549–56. 10.1177/039463201102400301 21978687

[8] Erridge C. Endogenous ligands of TLR2 and TLR4: agonists or assistants? J Leukoc Biol (2010) 87(6):989–99. 10.1189/jlb.1209775 20179153

[9] Hossain MJ Tanasescu R Gran B. Innate immune regulation of autoimmunity in multiple sclerosis: focus on the role of toll-like receptor 2. J Neuroimmunol (2017) 304:11–20. 10.1016/j.jneuroim.2016.12.004 28007303

[10] Xia Z White CC Owen EK Korff AV Clarkson SR McCabe CA Genes and environment in multiple sclerosis project: a platform to investigate multiple sclerosis risk. Ann Neurol (2015) 79(2):178–89. 10.1002/ana.24560 26583565 PMC4778957

[11] Giovannoni G Cutter GR Lunemann J Martin R Münz C Sriram S Infectious causes of multiple sclerosis. Lancet Neurol (2006) 5(10):887–94. 10.1016/S1474-4422(06)70577-4 16987736

[12] Buljevac D Flach HZ Hop WC Hijdra D Laman JD Savelkoul HF Prospective study on the relationship between infections and multiple sclerosis exacerbations. Brain (2002) 125(Pt 5):952–60. 10.1093/brain/awf098 11960885

[13] Andersen O Lygner PE Bergstrom T Andersson M Vahlne A. Viral infections trigger multiple sclerosis relapses: a prospective seroepidemiological study. J Neurol (1993) 240(7):417–22. 10.1007/BF00867354 8410082

[14] Edwards S Zvartau M Clarke H Irving W Blumhardt LD. Clinical relapses and disease activity on magnetic resonance imaging associated with viral upper respiratory tract infections in multiple sclerosis. J Neurol Neurosurg Psychiatry (1998) 64(6):736–41. 10.1136/jnnp.64.6.736 9647301 PMC2170117

[15] Mahadeva A Tanasescu R Gran B. Urinary tract infections in multiple sclerosis: under-diagnosed and under-treated? A clinical audit at a large University hospital. Am J Clin Exp Immunol (2014) 3(1):57–67. 24660122 PMC3960762

[16] Viglietta V Baecher-Allan C Weiner HL Hafler DA. Loss of functional suppression by CD4+ CD25+ regulatory T cells in patients with multiple sclerosis. J Exp Med (2004) 199(7):971–9. 10.1084/jem.20031579 15067033 PMC2211881

[17] Fletcher JM Lonergan R Costelloe L Kinsella K Moran B O’Farrelly C CD39+ Foxp3+ regulatory T Cells suppress pathogenic Th17 cells and are impaired in multiple sclerosis. J Immunol (2009) 183(11):7602–10. 10.4049/jimmunol.0901881 19917691

[18] Nyirenda MH Sanvito L Darlington PJ O’Brien K Zhang G-X Constantinescu CS TLR2 stimulation drives human naive and effector regulatory T cells into a Th17-like phenotype with reduced suppressive function. J Immunol (2011) 187(5):2278–90. 10.4049/jimmunol.1003715 21775683

[19] Nyirenda MH Morandi E Vinkemeier U Constantin-Teodosiu D Drinkwater S Mee M TLR2 stimulation regulates the balance between regulatory T cell and Th17 function: a novel mechanism of reduced regulatory T cell function in multiple sclerosis. J Immunol (2015) 194(12):5761–74. 10.4049/jimmunol.1400472 25980006

[20] Liew FY Xu D Brint EK O’Neill LA. Negative regulation of toll-like receptor-mediated immune responses. Nat Rev Immunol (2005) 5(6):446–58. 10.1038/nri1630 15928677

[21] Dulay AT Buhimschi CS Zhao G Oliver EA Mbele A Jing S Soluble TLR2 is present in human amniotic fluid and modulates the intraamniotic inflammatory response to infection. J Immunol (2009) 182(11):7244–53. 10.4049/jimmunol.0803517 19454721

[22] Raby A-C Le Bouder E Colmont C Davies J Richards P Coles B Soluble TLR2 reduces inflammation without compromising bacterial clearance by disrupting TLR2 triggering. J Immunol (2009) 183(1):506–17. 10.4049/jimmunol.0802909 19542461

[23] LeBouder E Rey-Nores JE Rushmere NK Grigorov M Lawn SD Affolter M Soluble forms of Toll-like receptor (TLR) 2 capable of modulating TLR2 signaling are present in human plasma and breast milk. J Immunol (2003) 171(12):6680–9. 10.4049/jimmunol.171.12.6680 14662871

[24] Iwami K-I Matsuguchi T Masuda A Kikuchi T Musikacharoen T Yoshikai Y. Cutting edge: naturally occurring soluble form of mouse toll-like receptor 4 inhibits lipopolysaccharide signaling. J Immunol (2000) 165(12):6682–6. 10.4049/jimmunol.165.12.6682 11120784

[25] Langjahr P Diaz-Jimenez D De la Fuente M Rubio E Golenbock D Bronfman FC Metalloproteinase-dependent TLR2 ectodomain shedding is involved in soluble toll-like receptor 2 (sTLR2) production. PLoS One (2014) 9(12):e104624. 10.1371/journal.pone.0104624 25531754 PMC4273945

[26] Perez J Dansou B Hervé R Levi C Tamouza H Vandermeersch S Calpains released by T lymphocytes cleave TLR2 to control IL-17 expression. J Immunol (2016) 196(1):168–81. 10.4049/jimmunol.1500749 26608921

[27] Polman CH Reingold SC Banwell B Clanet M Cohen JA Filippi M Diagnostic criteria for multiple sclerosis: 2010 revisions to the McDonald criteria. Ann Neurol (2011) 69(2):292–302. 10.1002/ana.22366 21387374 PMC3084507

[28] Lappin DF Sherrabeh S Erridge C. Stimulants of toll-like receptors 2 and 4 are elevated in saliva of periodontitis patients compared with healthy subjects. J Clin Periodontol (2011) 38(4):318–25. 10.1111/j.1600-051X.2011.01702.x 21284689

[29] Houssen ME El-Mahdy RH Shahin DA. Serum soluble toll-like receptor 2: a novel biomarker for systemic lupus erythematosus disease activity and lupus-related cardiovascular dysfunction. Int J Rheum Dis (2016) 19(7):685–92. 10.1111/1756-185X.12452 25123610

[30] Galliera E Drago L Vassena C Romanò C Marazzi MG Salcito L Toll-like receptor 2 in serum: a potential diagnostic marker of prosthetic joint infection? J Clin Microbiol (2014) 52(2):620–3. 10.1128/JCM.02727-13 24478497 PMC3911304

[31] Hasheminia SJ Zarkesh-Esfahani SH Tolouei S Shaygannejad V Shirzad H Hashemzadeh Chaleshtory M. Toll like receptor 2 and 4 expression in peripheral blood mononuclear cells of multiple sclerosis patients. Iran J Immunol (2014) 11(2):74–83. 24975964 10.22034/iji.2014.16768

[32] ten Oever J Kox M van de Veerdonk FL Mothapo KM Slavcovici A Jansen TL The discriminative capacity of soluble toll-like receptor (sTLR) 2 and sTLR4 in inflammatory diseases. BMC Immunol (2014) 15(1):55. 10.1186/s12865-014-0055-y 25406630 PMC4240815

[33] Metz L McGuinness S Harris C Urinary tract infections may trigger relapse in multiple sclerosis. Axone (1998) 19(4):67–70. 9849133

[34] Jaruvongvanich V Sanguankeo A Jaruvongvanich S Upala S. Association between *Helicobacter pylori* infection and multiple sclerosis: a systematic review and meta-analysis. Mult Scler Relat Disord (2016) 7:92–7. 10.1016/j.msard.2016.03.013 27237767

[35] Nichols FC Housley WJ O’Conor CA Manning T Wu S Clark RB. Unique lipids from a common human bacterium represent a new class of toll-like receptor 2 ligands capable of enhancing autoimmunity. Am J Pathol (2009) 175(6):2430–8. 10.2353/ajpath.2009.090544 19850890 PMC2789629

[36] Herrmann I Kellert M Schmidt H Mildner A Hanisch UK Bruck W *Streptococcus pneumoniae* infection aggravates experimental autoimmune encephalomyelitis via toll-like receptor 2. Infect Immun (2006) 74(8):4841–8. 10.1128/IAI.00026-06 16861672 PMC1539614

[37] Miranda-Hernandez S Gerlach N Fletcher JM Biros E Mack M Korner H Role for MyD88, TLR2 and TLR9 but not TLR1, TLR4 or TLR6 in experimental autoimmune encephalomyelitis. J Immunol (2011) 187(2):791–804. 10.4049/jimmunol.1001992 21685327

[38] Holst B Szakmany T Raby AC Hamlyn V Durno K Hall JE Soluble toll-like receptor 2 is a biomarker for sepsis in critically ill patients with multi-organ failure within 12 h of ICU admission. Intensive Care Med Exp (2017) 5(1):2 10.1186/s40635-016-0116-z 28092080 PMC5236041

[39] Zaharieva E Velikova T Tsakova A Kamenov Z. Reduced soluble toll-like receptors 2 in type 2 diabetes. Arch Physiol Biochem (2017) 21:1–4. 10.1080/13813455.2017.1401642 29160122

[40] Srinivasan S Su M Ravishankar S Moore J Head P Dixon JB TLR-exosomes exhibit distinct kinetics and effector function. Sci Rep (2017) 7:41623. 10.1038/srep41623 28290538 PMC5349571

[41] Hillman LJ Burns SP Kraft GH. Neurological worsening due to infection from renal stones in a multiple sclerosis patient. Mult Scler (2000) 6(6):403–6. 10.1191/135245800701566395 11212137

[42] Correale J Fiol M Gilmore W. The risk of relapses in multiple sclerosis during systemic infections. Neurology (2006) 67(4):652–9. 10.1212/01.wnl.0000233834.09743.3b 16870812

[43] Nicolle LE Uncomplicated urinary tract infection in adults including uncomplicated pyelonephritis. Urol Clin North Am (2008) 35(1):1–12. 10.1016/j.ucl.2007.09.004 18061019

[44] Khasriya R Khan S Lunawat R Bishara S Bignal J Malone-Lee M The inadequacy of urinary dipstick and microscopy as surrogate markers of urinary tract infection in urological outpatients with lower urinary tract symptoms without acute frequency and dysuria. J Urol (2010) 183(5):1843–7. 10.1016/j.juro.2010.01.008 20303096

[45] Hurlbut TA Littenberg B. The diagnostic accuracy of rapid dipstick tests to predict urinary tract infection. Am J Clin Pathol (1991) 96(5):582–8. 10.1093/ajcp/96.5.582 1951183

[46] Cook KW Crooks J Hussain K O’Brien K Braitch M Kareem H *Helicobacter pylori* infection reduces disease severity in an experimental model of multiple sclerosis. Front Microbiol (2015) 6:52. 10.3389/fmicb.2015.00052 25762984 PMC4327743

[47] Lina TT Alzahrani S Gonzalez J Pinchuk IV Beswick EJ Reyes VE. Immune evasion strategies used by *Helicobacter pylori*. World J Gastroenterol (2014) 20(36):12753–66. 10.3748/wjg.v20.i36.12753 25278676 PMC4177461

